# Barriers and facilitators to primary care for people with mental health and/or substance use issues: a qualitative study

**DOI:** 10.1186/s12875-015-0353-3

**Published:** 2015-10-13

**Authors:** Lori E. Ross, Simone Vigod, Jessica Wishart, Myera Waese, Jason Dean Spence, Jason Oliver, Jennifer Chambers, Scott Anderson, Roslyn Shields

**Affiliations:** Dalla Lana School of Public Health, University of Toronto, 155 College St. Suite 560, Toronto, Ontario Canada M5T 3M7; Social and Epidemiological Research Department, Centre for Addiction and Mental Health, 33 Russell St. Room T406, Toronto, Ontario Canada M5S 2S1; Women’s College Hospital and Research Institute, 76 Grenville Street Rm. 7234, Toronto, Ontario M5S 1B2 Canada; Department of Psychiatry, University of Toronto, 250 College St., 8th Floor, Toronto, Ontario Canada M5T 1R8; The Empowerment Council, 33 Russell St. Room 2008, Toronto, Ontario M5S 2S1 Canada; CATIE, 555 Richmond Street West, Suite 505, Box 1104, Toronto, Ontario M5V 3B1 Canada; Communications and Partnerships, Centre for Addiction and Mental Health, 100 Stokes Street, Toronto, ON M6J 1H4 Canada

**Keywords:** Mental health, Substance use, Primary care, Barriers, Facilitators, Qualitative research, Addictions, Collaborative models

## Abstract

**Background:**

Mental health and/or substance use issues are associated with significant disparities in morbidity and mortality. The aim of this study was to identify the mechanisms underlying poor primary care access for this population.

**Method:**

This was a community-based participatory action qualitative study, in which 85 adults who self-identified as having a serious mental health and/or substance use issue and 17 service providers from various disciplines who worked with this population participated in a semi-structured interview.

**Results:**

Client, service provider and health system barriers to access were identified. Client factors, including socioeconomic and psychological barriers, make it difficult for clients to access primary care, keep appointments, and/or prioritize their own health care. Provider factors, including knowledge and personal values related to mental health and substance use, determine the extent to which clients report their specific needs are met in the primary care setting. Health system factors, such as models of primary care delivery, determine the context within which both client and service provider factors operate.

**Conclusions:**

This study helps elucidate the mechanisms behind poor primary health care access among people with substance use and/or mental health issues. The results suggest that interdisciplinary, collaborative models of primary healthcare may improve accessibility and quality of care for this population, and that more education about mental health and substance use issues may be needed to support service providers in providing adequate care for their clients.

## Background

Mental health and/or substance use issues are common, affecting up to 1 in 5 individuals over their lifetime and represent a high burden of disease worldwide [1, 2]. Population-based studies have demonstrated a substantially increased risk of mortality for people with mental health and/or substance use issues compared to the general population that is three-fold for men and two-fold for women [3–5]. The Global Burden of Diseases, Injuries, and Risk Factors Study (2010) found that mental and substance use disorders were the leading cause of years lived with disability worldwide [2]. The causes of such disability are both mental health-related and attributable to medical causes such as diseases of the cardiovascular, respiratory and gastrointestinal systems [3].

However, treatment rates for these conditions are low around the world. A review of 37 studies in developed countries found that unmet need for mental health care is universally high, varying from at least 32 % for psychotic disorders to as much as 78.1 % for substance abuse problems [6]. Evidence suggests that under-treatment of individuals with mental health and/or substance use issues also extends to their medical health needs. For example, there is evidence of lower rates of cancer screening for individuals with mental health [7, 8] and substance use [9] issues, and there is also evidence that their medical illnesses may be undertreated [10, 11].

Primary care is a first point of contact and continuing point of care for many individuals with mental health and/or substance use issues. Yet, individuals with mental health and/or substance use issues report poorer access to and lower quality of the primary care received relative to those without [12, 13]. For example, in one survey of 59 patients of a community mental health centre, 14 % of participants reported that they did not have a usual source of primary care, and an additional 14 % reported using emergency medical services to address their primary care needs. In total, 63 % of this sample was unable to identify a primary care provider by name [14].

Some research has attempted to understand what factors account for these reported inadequacies in primary care delivery. For example, De Hert and colleagues [15] review a number of barriers to the recognition and management of physical diseases among patients with severe mental illness, including barriers related to the patient/illness, treatment, psychiatrist, other physician, and service. Chadwick and colleagues [16] review nine studies that have examined this issue from the patient perspective specifically, conducted in the US, United Kingdom, and Australia, and conclude that there are challenges associated with practical problems (e.g., financial and geographic accessibility) and interpersonal problems (e.g., between patient and provider, and between providers across disciplines). The authors note the need for further research on this topic from the service user perspective to “ascertain the global and specific barriers that prevent them from having their physical health needs addressed” (p. 218).

Much less is known about barriers to primary health care for people living with substance use issues, despite the fact that mental health and substance use conditions often co-occur. Research that does exist focuses primarily on the integration of treatment for substance use disorders within the primary care setting (see for example, [17, 18]). In this regard, it has been noted that treatment for substance use is much less integrated in primary care settings than is treatment for mental health issues [19]. We were unable to identify any prior research that has investigated service user perspectives on barriers to primary health care for individuals with substance use issues.

Our study, therefore, responds to Chadwick’s [16] call for more research in this area by extending existing knowledge about patient perspectives on barriers to primary care access to a) the Canadian setting, and b) individuals with substance use issues, in order to ultimately help inform interventions that can reduce morbidity and mortality for these populations.

## Methods

This was a qualitative, community-based participatory action research study [20]. Community-based participatory action research is a widely-used approach to health research that engages key stakeholders throughout the research process with the goal of creating meaningful change for the communities under study [21]. This project was conducted as a partnership between academic researchers, an organization representing clients of the mental health and addictions systems, and stakeholder organizations (including a large tertiary care mental health hospital, a family health team^1^, two community health centres, other community organizations serving individuals with mental health and/or substance use issues, and the provincial college of family physicians). Our primary research question was: *What are the barriers and facilitators to primary health care for people living with serious mental health and/or substance use issues?* In this study, we used the language “mental health and/or substance use issues” in reference to conditions associated with mental health, the use of substances, or the combination of the two that have impacted an individual’s quality of life. We used this language in contrast to language such as “psychiatric disorder” or “addiction” at the request of our community partners, in order to a) respect individuals as the authorities to define for themselves whether their mental health/substance use is problematic or requires intervention, b) acknowledge the harm that has sometimes been done to individuals and communities through the application of diagnostic labels, and c) be inclusive of factors situated outside of the individual (e.g., discrimination, poverty) that often coexist with and have significant impacts on individuals’ mental wellbeing and/or use of substances. In this paper, we use other language only where necessary to be consistent with the authors of the original studies cited.

All study procedures were reviewed and approved by the Research Ethics Board of the Centre for Addiction & Mental Health, a tertiary care hospital fully affiliated with the University of Toronto, Canada. Client participants were recruited via distribution of flyers through the client advocacy and stakeholder partner organizations. Eligible client participants were 18 years of age or older, sufficiently fluent in English to understand the consent form and participate in an interview, and self-identified as having a serious mental health and/or substance use issue. If potential participants asked for clarification regarding this latter criterion, they were provided with the following definition: “We are looking for people whose mental health and/or substance use issues have impacted their quality of life, although they may have experienced periods of recovery or well-being. Participants may have been diagnosed with a psychiatric condition and/or an addiction, although this is not a requirement.” In total, 102 clients were screened, of whom 96 were eligible (3 did not self-identify as having a serious mental health or substance use issue, and 3 were not able to provide informed consent). Of the 96 eligible individuals, 10 could not be re-contacted, 86 were invited to participate in a face-to-face 30 minute interview, and 85 consented and completed an interview.

Service provider participants were also identified through partner organizations. Each organization was asked to nominate 1–2 service providers of any discipline who they felt had knowledge pertinent to the research question. All 17 selected service providers agreed to participate.

MW, DS and SA conducted interviews with 85 clients between September 2011 and October 2012, and with 17 service providers between October 2011 and August 2012, using a semi-structured guide (available upon request) that had been field-tested with members of the research team who identified with the participant groups (i.e., research team members who were themselves service users or providers). Following the interview, client participants received a cash honorarium plus compensation for public transit; clients were also provided with a snack and a drink during the interview. Service providers received a voucher towards the purchase of mental health/substance use related resources from the publishing department of the primary research institution.

### Data analysis

Interviews were digitally recorded and transcribed verbatim, except in 3 cases where participants requested not to be recorded, and 2 cases of equipment failure. With the exception of one interview for which the equipment failure was identified too late for reliable note-taking to occur, analysis of these interviews was based on hand-written notes taken by the interviewer immediately following the interview (i.e., when the recording failure was identified). Each transcription was reviewed by the interviewer or another member of the project team (other than the transcriptionist) for verification.

De-identified transcripts were then analyzed using a modified grounded theory approach [22, 23]. According to Glaser and Strauss [22], grounded theory is an analytical approach that allows for the “discovery” of theory that is grounded in data on a given topic. Central to a grounded theory analysis is the constant comparative method, wherein data analysis is undertaken concurrently with continued data collection, in order to allow for continuous comparison of data to emerging categories [23]. In this manner, the interview guides could be amended to address key themes and explore emerging theories identified in preliminary analysis [24]. Transcripts of client interviews were analyzed first, followed by transcripts of service provider interviews.

The usual process of grounded theory analysis was modified slightly for consistency with the community-based participatory action approach of this project. Specifically, we identified particular phases of the data analysis process that would be most feasible and appropriate for the integration of perspectives from our various stakeholder partners. The first stage of our analysis involved collaborative development of a preliminary coding framework. Specifically, the interviewers selected six client transcripts which they believed to reflect many of the key topics and ideas emerging from the interviews that had so far been conducted. These interview transcripts were then independently analyzed by several members of the research team (including 6 of the authors, and with representation of academic, service user, service provider, and policy partners), using an open coding procedure wherein any text in the interview that the analyst perceived to be relevant to the primary research question (i.e., reflecting barriers or facilitators to primary health care access) or that was repeated within and between interviews was identified and labeled as a code (e.g., “open-minded”). The analysts then met to compare and contrast their open coding of these initial interviews, and on the basis of their insights, applied the principles of axial coding [23] to establish a preliminary coding framework. This coding framework organized the barriers and facilitators to primary care into themes (e.g. “provider values and attitudes”) at the client, service provider, and health systems levels. Themes were considered for inclusion in the coding framework when they were directly relevant to the research question and either: a) appeared repeatedly across many of the interviews (including both comparative (e.g., judgmental attitude) and contrasting (e.g., non-judgmental attitude) experiences related to the theme), or b) described elements of primary care access that were central to the experiences of some participants. Themes were agreed upon through consensus between all analysts. MW, JW, SA, LR and DS then coded the remaining transcripts on the basis of this framework (with at least 2 independent analysts for each transcript). The independently coded transcripts were compared and any disagreements resolved through discussion with the principal investigator (LR) to agree upon a final coded version of each transcript which was entered into NVivo software (NVivo qualitative data analysis software, QSR International Pty Ltd. Version 9, 2010) for the purposes of data management. The coding framework was revised and refined as needed through this process. For example, analysis of the service provider interviews required revision to the coding framework to add themes for health systems barriers and facilitators that had not been mentioned in the client interviews. After each revision to the coding framework, previously coded interviews were reviewed and the coding adjusted as required.

**Fig. 1:**
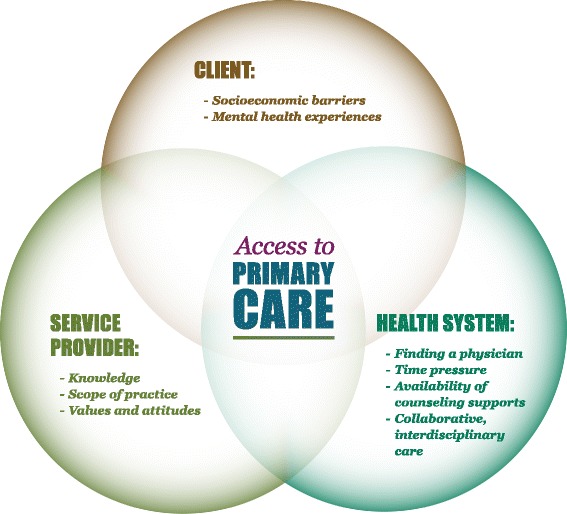
Barriers and facilitators to primary care access for people living with mental health and/or substance use issues: Ontario, Canada, 2011–2012

Following the coding procedure, the coded text for each theme was reviewed and summarized into theme memos by MW, JW, DS and JO. These memos were first reviewed by LR for fidelity to the data (i.e., to ensure that each of the key arguments in the memo could be strongly supported by direct quotations from the data set). Finally, the entire collaborative research team reviewed and discussed the theme memos in order to come to consensus upon our final theoretical model of barriers and facilitators to primary care access for individuals living with mental health and/or substance use issues (as illustrated in Fig. 1).

## Results

Participant characteristics are provided in Tables 1 and 2. Clients were predominantly Canadian-born, had annual household incomes less than $20,000 CAD, and self-identified as having a depressive, anxiety, or schizoaffective disorder. Fifty-one (60 %) of participants self-identified as having a substance use issue, either alone or in combination with a mental health issue. Substance use in this sample predominantly related to use of alcohol, opiates, or crack. Service providers reflected a variety of disciplines (predominantly nursing/nurse practitioners and social work) and types of services (predominantly hospital inpatient, hospital outpatient, and community health centres).

**Table 1:** Characteristics of client participants in a qualitative study on access to primary care

	*N* (%) or Mean (range)
Gender identification (*N* = 84)	
Female	45 (52.9)
Male	37 (43.5)
Transgender	2 (2.4)
Age	43.0 (20–83)
Place of birth	
Canada	70 (82.4)
Outside Canada	15 (17.6)
Relationship status	
Married, Common-law or Equivalent	19 (22.4)
Separated, Divorced, or Widowed	17 (20)
Single	45 (52.9)
Other	4 (4.7)
Type of dwelling (*N* = 84)	
Stable Housing (shared dwelling, own home, subsidized home)	58 (69.1)
Unstable housing (shelter, boarding home, transitional, other)	26 (31)
Co-habitation status	
Live-in partner/spouse	18 (22)
Alone	25 (29.4)
Source of income (*N* = 84)^a^	
Full-time employment	3 (3.6)
Part-time employment	12 (14.3)
Family Support	5 (5.9)
Disability (ODSP, CPP-D, WSIB, Private)	47 (55.9)
Social Assistance: OW	25 (29.8)
Pension	4 (4.8)
Yearly household income	
≤ $20, 000	60 (70.6)
$21,000-$35,000	13 (15.3)
≥$36,000	7 (8.2)
Unknown or did not wish to disclose	5 (5.9)
Highest level of education	
Less than or some high school	21 (24.7)
Completed high school	10 (11.8)
College or University (some or completed)	46 (54.1)
Post Graduate (some or completed)	8 (9.4)
Self-identified mental health issue (selected)^a^	
Depression	34 (40.0)
Anxiety	21 (24.7)
Schizophrenia/Schizoaffective disorder	17 (20.0)
Post-traumatic stress disorder	13 (15.3)
Self-identified substance use issue (selected)^a^	
Alcohol	21 (24.7)
Opiates	12 (14.1)
Crack	10 (11.8)

^a^Participants could select more than one category, therefore frequencies do not total 100 %

*N* = 85, unless otherwise specified

**Table 2:** Characteristics of service provider participants in a qualitative study on access to primary care

	*N* (%) or Median (range)
Gender identification	
Female	15 (88.2)
Male	2 (11.8)
Age (*N* = 16)	38 (26–57)
Type of service	
Hospital In-Patient	6 (35.3)
Hospital Out-Patient	5 (29.4)
Community Health Centre	5 (29.4)
Private Practice	1 (5.9)
Other Community-Based Program	1 (5.9)
Other	2 (11.8)
Professional discipline	
Nurse	1 (5.9)
Social worker	4 (23.5)
Psychiatrist	2 (11.8)
Family physician	2 (11.8)
Case manager	1 (5.9)
Nurse practitioner	3 (17.6)
Peer recovery facilitator	2 (11.8)
You don’t have an option that applies to me^a^	2 (11.8)

^a^Included: counselling psychologist, mental health counsellor

*N* = 17, unless otherwise specified

In the sections that follow, we use illustrative quotations from both client and service provider participants to describe the major themes identified at each of the client, service provider, and health systems levels. Participants have been assigned pseudonyms for the purposes of protecting their confidentiality.

### Client-level factors

Two primary barriers operating at the client level were identified in our data: 1) socioeconomic barriers, and particularly those associated with poverty, unstable housing, and related barriers to access; and 2) barriers related to mental health experiences and side effects of the medications or other substances client participants were using.

#### Socioeconomic barriers

Both client and service provider participants spoke extensively about the serious impact of socioeconomic barriers, and particularly those related the impact of poverty and associated unstable housing and lack of transportation, on access to primary care. As noted in Table 1, the majority of client participants in this study were living on disability or other income support, and as such, had very minimal incomes to cover costs associated with housing, food and other necessities. In this context, many client participants experienced unstable housing and did not have a fixed address or telephone number:

“And it’s hard ‘cause I mean you call and leave a message [at the physician’s office], and then, especially in my situation right now not having a phone or anything like that, you can’t leave a number here.” (Gianna, age 27, has a regular provider)

“I think it’s hard anyway to get a family doctor, so it’s even harder to get a primary care provider if you don’t have a phone, if you don’t have a number where they can reach you. If you’re very transient. If you’re not living in one place.” (Whitney, nurse practitioner)

Both clients and service providers perceived that it was challenging to find and maintain a family physician in the context of unstable housing. The client’s lack of funds to cover costs of transportation in order to visit a primary care provider was also identified as a barrier. These practical barriers determined the extent to which clients were able to make their primary care a priority relative to more acute issues such as housing and food security:

“We need to do some of the basics, with housing, shelter, food. Finances….Without that basis, it’s difficult for clients to get better. Because they have so many other worries, about their housing, and their food and whatever. How can you concentrate on your health when you’re just trying to survive?” (Kim, nurse practitioner)

“I tried even to get blood tests or something… they go, ‘What? You don’t have a health card so you’re gonna have to pay cash for it.’ So then never mind, ‘See you later’ and then I leave.” (James, age 53, has a regular provider)

#### Mental health experiences and side effects

Both clients and service providers spoke about the impact of clients’ mental health experiences on their capacity to access primary care:

“There were times when I was depressed and missed appointments, but it would be my fault. I just wouldn’t feel like getting out of bed. I was really, really sick and so forth. I’ve had many times that I’ve skipped appointments because I wasn’t feeling well, mentally.” (Judith, age 67, has a regular provider)

In particular, both client and service provider participants described the challenges that anxiety or psychosis can present in the context of a crowded waiting room environment or in the face of needed medical tests, particularly when these involved lengthy wait times or early morning appointments (in that mental health and/or substance use problems are often associated with sleeping difficulties and/or use of medications that make waking difficult):

“[Clients] get more nervous as they’re waiting to see the psychiatrist or the doctor, so they start to have that conversation with that voice. It can scare people. I’ve had someone else who was even told for awhile that they might lose their access to the health centre because they were acting out in the waiting room, because they had such high anxiety they would start literally crying and screaming in the waiting room because they couldn’t wait to see the doctor.” (Jasmine, case manager)

Clients and service providers also highlighted the impact of side effects—associated both with prescribed medication such as sedatives and with non-prescription substance use—on client capacity to make and keep appointments:

I can make appointments to see [a primary care provider at the community health centre where he was interviewed] but people with mental issues and addictions-- I have a very severe addiction. I’m a crack addict. For me to sit here for a half an hour right now, it’s killing me. (Barry, age 51, does not have a regular provider)

These experiences often made it difficult for people to keep appointments, resulting in frustration for both clients and service providers, and often avoidance or delay of needed care.

#### Provider-level factors

Our data indicate that participants perceived primary care providers’ capacity to address the needs of patients with mental health and/or substance use issues to be determined by three key factors: (1) their knowledge related to mental health and substance use; (2) their willingness to address these issues within the scope of their primary care practice; and (3) their personal values and attitudes in relation to mental health and substance use, particularly as participants perceived these to determine providers’ capacity for delivering health care in an empathic and person-centred manner.

#### Knowledge

Both client and service provider participants perceived that some primary care providers lacked adequate knowledge about mental health and/or substance use issues:

“[Walk-in clinic physicians] don’t [address addictions issues] because number one they’re not trained for it, you know. Number two, they wouldn’t really know where to send you.” (Arlene, 54 years old, no regular provider)

“If somebody has a mental health issue, if somebody just has schizophrenia, people are frightened. People don’t know enough about it. They don’t have enough education – they feel like it would be too complicated to manage.” (Whitney, nurse practitioner)

As a result of this perceived lack of knowledge, one of the primary recommendations made by both client and service provider participants was for education of primary health care providers:

“It’s education, mainly – the doctors and nurses need to be educated about mental health, because for many of them, they are like ‘We don’t know about your situation. We don’t know what’s wrong with your mind.’ (Taban, 52 years old, has a regular provider)

#### Scope of practice

Client participants reported that many primary care providers seemed unwilling to engage in conversations about mental health or substance use issues, explicitly or implicitly sending clients the message that these concerns were outside of the scope of their primary health care practice:

“Well, you know, it’s a kind of subtle message that says ‘well, look. I am here for your physical health’ and they don’t look at the big picture – how the mental impact on the physical, you know, and that we are a whole…‘I can’t help you, sorry. I want to help you, but my area, is the body.’” (Gyala, age 62, has a regular provider)

Not all client participants felt it was necessary for their primary care providers to see their mental health and/or substance use issues as within their scope of practice, however, so long as they could make appropriate referrals:

“He’s concerned about my well being, mental health and my physical health. Mentally – he’s the one they referred me to [tertiary care mental health hospital]. If it’s something that he sees that’s outside of his scope as an MD [family doctor] – that’s what I like about him. He looked for other sources, in a more specialized area. Here I could see a psychiatrist or someone practicing psychiatric medicine here. And that’s out of his scope. And he’s humble enough to know that and refer you to someone else. Because he wants the best for me, I think.” (Judith, age 67, has a regular provider)

Service provider participants also shared the perception that many primary care physicians saw mental health and substance use issues as outside of the scope of their practice, and connected this back to their perceived lack of knowledge about these topics:

“I don’t know what doctors get, but from a nursing and an NP [nurse practitioner] point of view, psychiatry and addictions are a very small part of the course curriculum, so really, you come out with no knowledge. And there’s this push to have our clients integrated in community and receive their services in community, but if you don’t have practitioners who are really well educated in those issues, then how can you provide the service? And if you don’t feel that you have the knowledge to provide care, obviously, you’re going to back off, or be anxious about or reluctant to provide care to that type of client, because you don’t feel skilled enough.” (Kim, nurse practitioner)

#### Values and attitudes

Client experiences were profoundly impacted by the values and attitudes of the primary care providers they encountered:

“If you go to your own family doctor—unless he’s very open-minded--you’ll be shamed out of there, you know. You certainly can’t go to a walk-in clinic and mention [substance use] because they call it drug-seeking.” (Arlene, age 54, does not have a regular provider)

Provider stigma was particularly problematic for participants with substance use issues, though many clients who reported only mental health issues also spoke to this barrier:

“I said ‘I have DID [dissociative identity disorder]’ and she [health care provider] said, ‘What’s that?’ And I [told] her and she stopped the interview right there and said ‘We don’t deal with people like you here’….So I just left and started crying, and walked home.’” (Lanette, age 34, has a regular provider)

In many cases, mental health stigma was deeply interconnected with other forms of discrimination, and particularly stigma related to poverty, homelessness and criminalization:

“I can’t access anybody…There is no access. I don’t have money. If I had money… if I was, you know, a politician, there’d be lots of access. If I was working a straight job, there’d be lots of access. But because I’m an ex-convict, whose got mental health and addiction problems, there’s nothing for me.” (Garret, age 47, does not have a regular provider)

However, of the client participants who reported having a regular primary care provider (approximately 80 % of our sample), many had very positive relationships with them. These positive relationships seemed to be characterized by providers who delivered care in an empathic and person-centred manner. Specifically, clients described providers who were able to look beyond their diagnoses and see them as whole people, with a variety of physical health and psychosocial needs, many unrelated to their mental health and/or substance use issue—but also able to appreciate the multitude of ways in which their mental health and/or substance use issue affected their lives:

I like the fact that he [family doctor] takes a personal interest in me as his patient. He’s a very professional doctor, but he also goes a little bit beyond that, in terms of showing interest. He will want to know how you’re doing, and he takes the time to hear something that’s not relevant or whatever. You’re just saying something that took place in your life. He’ll call, on the phone too – ‘I haven’t seen you this week, is everything ok?’ and it’s just a matter of checking up. It’s wonderful. (Judith, age 67, has a regular provider)

Non-judgmental treatment on the part of health care providers was made all the more meaningful for individuals who used substances and faced discrimination on a daily basis:

“On the lighter side there’s also really, really good ones. They’ll say, ‘You know what dear? I can see you’re addicted to drugs or opiates but hey, you know what? I’m gonna try to help.’….The ones that are non-judgmental; they take your breath away.” (Francesca, age 52, has a regular provider)

“Sometimes when I’m not in my right head, if I’m a little intoxicated, she talks to me like, you know, like a human being, and that’s what I respect about her.” (Aurelie, age 53, has a regular provider)

As a result of these relationships, clients felt cared for and empowered to take the steps necessary to care for their own physical health. This participant describes her physician’s role in supporting her to quit smoking:

“[The doctor] didn’t lecture me. He didn’t make me feel bad. He just said ‘Well, I know you’re a smart girl’ and he kind of gave me credit for knowing better. And he said ‘this is what I want you to do—and if it’s not working out, I want you to call me. Either way, I want to see you back here in 6 months and we’ll take it from there.’ And in 6 months, I had actually stopped smoking.” (Keeya, age 44, has a regular provider)

These interactions seemed to be very much related to provider values and attitudes. Providers who were able to see clients as whole people, and not simply a diagnostic label, were seen by both provider and client participants as key facilitators of primary care access for this patient population.

#### Health system-level factors

Participants also described systems-level barriers to accessing primary care that have unique and often substantial impacts on people living with mental health and/or substance use issues. Particularly relevant barriers were: challenges in finding a regular family physician; having all health needs addressed in a timely manner; lack of availability of counseling/support groups; and barriers associated with models of primary care.

#### Finding a physician

Participants perceived that several factors exacerbated difficulty in finding a consistent family physician for individuals with mental health and substance use issues. These included newcomer status, substance use, mental health symptoms, poverty, housing instability and transience, complex care needs, physical disability, and criminal records. Increased paperwork load for bureaucratic processes associated with physician assessments that are required for financial disability support and other forms of social assistance was also a barrier. While client participants described a general perception that they were undesirable patients because of their mental health and/or substance use issues, service provider participants perceived that the factors listed above contributed to a general unwillingness for practitioners to take on patients who experience complex care needs.

“No availability of doctors…sometimes when you make the referral, they are turned down because they are ‘too complex’. Their addictions and their mental illness are [perceived by the doctor to be] just too complex.” (Linda, nurse practitioner)

#### Time pressure

Interviewees identified barriers involved in making and keeping appointments with physicians. Service users spoke to the lengthy wait times involved in securing an appointment with a primary care provider.

“Sometimes, I make an appointment, and sometimes I just go to the drop-in because making an appointment is a sense of hassle. You have to wait 2 weeks, 3 weeks, things like that.” (Norine, age 33, has a regular provider)

Client participants expressed frustration with only being able to have one healthcare concern addressed at a time or feeling rushed through appointments with primary care providers. Service providers also stated that the time allocated to each appointment was not sufficient to address healthcare needs for this population.

“Facilitators from the clinician’s perspective would be to have more time for each client. And not being so rushed…I don’t even have enough time to check in with a client. And I know that if I had more time to divide it up between more people more evenly, I would find issues much quicker.” (Magda, social worker)

Alternatively, some client participants shared affirmative experiences regarding the time allotted to their appointment, such as their physician taking the time to respectfully address and acknowledge their concerns. Some clients also spoke positively about the prompt service they received in securing an appointment. Other appointment facilitators mentioned were reminders, a knowledgeable and responsive reception staff, and consistency of service provider hours, especially in drop-in settings.

“This idea of the same day every week…I tell my clients “Same Bat Time, same Bat Channel’ …I try to have the same time for the appointment, same day of the week, and I have the same office day so you can always find me.” (Jasmine, case manager)

#### Availability of counseling supports

Many client participants felt that they were not receiving adequate support for their emotional health. They felt that physicians did not (or were not able to) provide information about the availability of these supports, nor help to connect clients to these services. They also felt that there were inadequate services following in-patient treatment when more intensive support was required, and that high provider turn-over limited benefit from counseling services when they were available. Client participants expressed frustration with what was perceived as a “rush” to prescribe medication, despite client preference for ‘talk therapy.’ Clients also faced long waitlists to see psychiatrists (some of whom are covered under universal health care in Canada, while others charge additional fees) and financial barriers to accessing therapy with other mental health services not covered by provincial health insurance.

“There’s nowhere to go in this city that you can sit down and talk to somebody when you have an issue…there’s no place that has counsellors that you can go sit down and talk to. A nurse practitioner’s fine, but they don’t understand mental health issues.” (Garret, age 47, no regular provider)

Both clients and service providers stressed the importance of support workers in their healthcare. This support included helping connect clients to services, managing appointments, and in some cases, accompanying clients to appointments.

“They [support workers are] really taking good care of me. They really help me. Especially [Case Manager A] – the first time I came, because I was so scared. I was nervous. I felt, you know, ‘I’m not safe, even here’. Because I was really unsafe at home. So I was so traumatized. I had nightmares and stuff. And she took it upon herself to take me to a psychologist. People who can help me mentally. And then she took me to this [Health Centre], and I met [Counselor A]. She never even told me to go to [Health Centre] – she took me there herself, you know.” (Miyanda, age 35, no regular provider)

#### Collaborative, interdisciplinary care

Clients expressed frustration with needing to access care for physical health, mental health and social concerns from a number of different locations. Service users and providers emphasized their preference for models of care where physical, mental health and social service concerns could be addressed in one location to improve communication between providers. Service providers in particular felt these models increase the quality of care for individuals with complex health needs.

“I like the fact that [Community Health Centre] is a one stop shop. So you can see a nurse. You can see a lab tech. You can see a physician. They’ll make referrals within their system.” (John, age 55, has a regular provider)

“I think that addressing people’s needs, not just specific to one particular issue, but trying to connect with all the issues they’re going through [is helpful]. People’s problems are multilayered and I think family health teams1 do a good job of that. They’re also in environments that aren’t necessarily alienating, like the places where they’re located aren’t as institutional as coming to a large hospital, or going to a walk-in clinic or something like that, where you feel it’s very rushed. I think it has that…interdisciplinary aspect that’s important to people.” (Aria, social worker)

Service providers spoke to the lack of communication and collaboration between health professionals as a major barrier to quality care for people living with mental health and substance use issues. This lack of communication occurred between institutions, between healthcare disciplines (e.g. physicians, nurses, other allied health professionals), between providers delivering mental and physical health services, and in the transition to community-based care after psychiatric hospitalization.

“So I would say that sometimes, the communication is not the greatest between (Hospital name) and the community providers. And from a psychiatric point of view, I don’t know that psychiatry communicates very well with the community GP. I would be doubtful on that issue. Communication could probably be better than it is. Sometimes it’s a little hard to get the information that you need from the practitioners and the community.” (Kim, nurse practitioner)

Service providers in particular felt that interdisciplinary, collaborative models of primary care delivery (e.g., family health teams, community health centres) increased the quality of care for individuals with complex health needs, not only by addressing communication challenges, but also by making primary health care more comprehensive and feasible for these clients to access.

## Discussion

We have identified barriers and facilitators to primary care access at the client, provider, and health system levels which help to elucidate the mechanisms behind the high rates of preventable morbidity and mortality among individuals living with mental health and/or substance use issues. Specific client factors, including practical and psychological barriers such as poverty and an inability to tolerate long waiting room times, make it difficult for clients to access primary care, keep appointments, and/or prioritize their own health care. Provider factors, including knowledge and personal values related to mental health and substance use and ability to deliver health care in an empathic and person-centred manner, determine the extent to which clients feel their specific mental and physical health needs are met in the primary care setting. Finally, health system factors, such as provider availability and models of primary care delivery, determined the context within which both client and service provider factors operated. Together, barriers at all three levels made it very difficult for individuals living with mental health and/or substance use issues to access and maintain quality primary care.

These findings are consistent with the little available literature that has examined primary health care access for this population, despite different geographic settings and in turn, different health care systems. For example, Lester et al. [25] conducted a qualitative focus group study with individuals diagnosed with “serious mental illness” and their primary care providers in six primary care trusts in the United Kingdom. Their findings indicated that primary care is considered central to health care by these service users, but that both timely access to primary care providers and reluctant involvement on the part of some providers present significant challenges.

The primary strength of our study is its diverse sample, particularly with respect to representation of low socioeconomic status and individuals with substance use issues. As a qualitative study, our findings are not intended to be generalizable to the broad population of individuals living with mental health/substance use issues. However, it is appropriate to consider whether there are other contexts within which our key findings regarding barriers and facilitators to primary care are likely transferable. In this regard, we would remind the reader that all of our participants were living in urban areas in Canada, and as such, the barriers and facilitators described here may not be transferable to other countries and/or smaller communities where health care services may be structured differently. Further, our data were collected from participants who were connected in some way to publicly funded, community services, and as such, may not reflect the experiences of individuals who are able to pay to access services that are also available privately (such as counselling). Related to this, all of our participants had some attachment (though often tenuous and/or fragmented) to one of the participating service organizations. As such, the experiences of individuals who are completely marginalized from the health and social service systems may not be addressed by this study. Finally, client participants self-selected into this study; there was no purposeful sampling of client participants. It is possible that individuals motivated to participate in a research study on this topic may have different experiences or perceptions than those who were not motivated to do so.

The results of this study have a number of implications for health care research, practice and policy. First, our study offers implications for provider education, as many of the participants in this study felt that the primary care providers they had accessed lacked knowledge about mental health and/or substance use, and also had high levels of stigma regarding these issues. These findings are consistent with other research which has indicated that family physicians themselves feel insufficiently trained on these topics [26]. Additional research to determine if this is also the case for providers educated elsewhere is warranted, and if so, education for providers to address these gaps is therefore essential to enable delivery of quality primary care to this population. Indeed, there are already some promising models for integrating issues related to mental health and substance use into the training of primary care providers [27].

Further, the results of this study suggest that some individuals with mental health and/or substance use issues prefer interdisciplinary, collaborative models of primary health care, in which services to address physical health, mental health, substance use, and social determinants of health are offered under one roof, and further, that models of care that lack such integration may present barriers for these populations. This is consistent with other research: a substantial number of reviews and meta-analyses have established the effectiveness of collaborative care for the treatment of depression [28–31]. In addition to this robust evidence for depression, a review of randomized controlled trials and high quality quasi-experimental studies found some evidence for benefits of integrated care models on anxiety, attention deficit-hyperactivity disorder, and at-risk alcohol use [32]. Although there is a less robust evidence base, some studies have also found improvements in physical health outcomes associated with collaborative models of care [33]. For example, a randomized trial examining the impact of a medical care management intervention on individuals with ‘serious mental illness’ found that the intervention group reported significantly higher rates of preventive health screenings than the non-intervention group [34]. Another study examined the potential impact of colocation of general medical care within Veteran’s Affairs mental health treatment settings in the US, and found better outcomes on four of nine physical health indicators in those clinics with collocated services [35]. In response to this body of evidence, various professional associations have called for better integration of mental health and substance use care within primary health care [36, 37].

However, broadening the availability of such models of care requires consideration of primary health care funding. Both client and service provider participants in this study made direct links between inability to secure a family physician and perceived complexity of patient needs, and service providers highlighted current funding models as a major contributor to physician unwillingness to take on these patients. Although traditional fee-for-service models are not conducive to addressing the barriers described here, capitation funding models may also be problematic [38, 39], in that primary care settings operating under capitated models may de-roster patients that use outside primary care and in so doing, drop patients with higher health needs. Alternative solutions might include incentives, capitation models based on diagnoses, salaried models, funding of non-physician primary care providers, or funding models based on community needs [40]. Research is needed to determine which approach is optimal to meet the needs of people living with mental health and/or substance use issues, in order to address the significant disparities in morbidity and mortality for this population.

## Conclusions

This study has identified client, service provider and health systems level barriers to primary care access for people living with mental health and/or substance use issues. Interventions to address these identified barriers at the client, service provider and health system levels will collectively improve the accessibility and quality of primary health care for individuals living with mental health and/or substance use issues, which in turn may reduce associated disparities in morbidity and mortality. Specifically, the results suggest the need for future research to investigate the potential value of interdisciplinary, collaborative models of primary health care for this population, as well as the need for primary care provider education in the areas of mental health and substance use.

## Footnotes

1 Family Health Teams (FHTs) are the most prevalent model of shared primary care in Ontario, Canada (the setting for this research). FHTs include a variety of health professionals; commonly primary care physicians, nurses and nurse practitioners, dieticians, and social workers. FHTs are often focused on chronic disease management and prevention, community health needs and health promotion. (https://www.ontario.ca/page/family-health-teams).
